# Virtual screening workflow for glycogen synthase kinase 3β inhibitors: convergence of ligand-based and structure-based approaches

**DOI:** 10.1186/1758-2946-3-S1-P17

**Published:** 2011-04-19

**Authors:** VA Palyulin, DI Osolodkin, NS Zefirov

**Affiliations:** 1 Department of Chemistry, Moscow State University, Moscow, 119991, Russia

## 

Glycogen synthase kinase 3β (GSK-3β) is a serine/threonine protein kinase participating in a number of cellular pathways (e. g., insulin and Wnt pathways). Inhibitors of this kinase show antidiabetic properties, may be used for the treatment of Alzheimer's disease and neuroinflammation, have memory-stabilizing effect. The unique combination of various effects in the same target makes the kinase very attractive for the design of new perspective drugs.

A computational search for ATP-competitive GSK-3β inhibitors in ZINC database was performed with the use of two complementary methods: structure-based screening (docking with FRED) and ligand-based screening (shape similarity search with ROCS). ZINC database was prefiltered according to rules of lead-likeness; most notably, it was forced for potential inhibitors to possess at least one ring structure with the aim to restrict flexibility of the molecule and make it more synthetically accessible. Comparative analysis of the applicability of various scoring functions to structure-based search for GSK-3β inhibitors was carried out. The best performance was observed for the functions based on the complementarity between the ATP-binding cavity of the kinase and inhibitor molecule (Shapegauss, Chemgauss3, Chemscore). The structure-based screening system was evaluated with the help of Receiver Operating Characteristic (ROC) analysis to assess the overall enrichment of the database and BEDROC metrics for early recognition of the hits. After visual inspection of docking results, 2500 reasonable hits were identified, some of them belonging to previously unexplored structure classes.

Ligand-based search was performed using the structures of cocrystallized GSK-3 inhibitors available in PDB as a template. The optimal model was chosen according to the ROC analysis; optimization of the model included the removal of excessive features and weight increase for essential features. Similarity search was performed for the same ZINC subset as docking. Five thousand hits were identified, and docking was performed to compare them with the hits found during the structure-based search. Despite the fact that ligand-based screening was not constrained by the requirement for the presence of certain hydrogen bonds patterns, certain scaffolds appear in both hitlists. We may conclude that the sequential use of ROCS and FRED may be considered as a quick way to identify hits, whereas docking of large databases should be used when chemical diversity is needed to be explored.

